# Regular Meeting of the Atlanta Society of Medicine

**Published:** 1897-08

**Authors:** 


### REGULAR MEETING OF THE ATLANTA SOCIETY OF MEDICINE.

ATLANTA, GA., June 15, 1897.

The meeting was called to order by the President, Dr. G. H. Noble.

The following members were present: Drs. Benson, Crawford, Divine, Duncan, Longino, Murphy, Robinson, Noble, Hancock, Visanska, McRae, Stirling, Love.

The minutes of the previous meeting were read and approved.

Dr. Kendrick had been appointed to read a paper, but was unable to be present.

REPORT OF CASES.

DR. CRAWFORD: I have a patient here upon whose nose I operated. As you can not see in what condition her nose was before operative measures were taken, I have brought with me these photographs, which were taken just previous to the operation. The case was brought to me last June. She was in a very deplorable condition, as destruction of the nose had been going on for some months. The cartilage, bones and turbinated bodies were all gone and the integument had sloughed away, leaving only the septum and the tip of the nose. My idea was to get her well, and I will say nothing at present about the cause of this condition. I began treating her June 1st and continued until September and then made the first operation. On the photograph you will notice that the opening was very large and I had to fill that up to make a protection. I treated her from June to September with the iodides or mixed treatment. She went up to 100 drops of the saturated solution three times a day, and is now taking about fifty drops. On September 22d, under ether, I made the operation. I prepared the edges of the opening all around, and then with a sharp bistoury I made an incision upward between the eyebrows, across the forehead, nearly to the hair, then along the forehead about two and a half inches, then back down the nose. This gave a triangular piece of integument attached by a small pedicle. I took this integument and twisted it upon itself and stitched it over the opening. Union was very good, and at the end of a couple of weeks I cut the pedicle, straightened it out, and put it back into position. This was done two weeks after the 22d of September. Now, two weeks ago I made another operation. You will notice here on the right side there is a little point that I brought from the uneven side of the nose. I found no trouble in getting the parts to grow together; the flap from the forehead grew well enough, but it dropped suddenly and caused the nose to turn up. So, two weeks ago the apex of this V-shaped piece being above, I brought it across the nose and the nose is now somewhat straight. Later on I expect it to be even better than it is now.

DR. STIRLING: I have nothing specially to say on the subject. I think both the Doctor and the patient should be pleased with the result. Often these cases are very unsatisfactory, and I think in this one the results are very good.

DR. BENSON: Dr. Crawford told me that in this case he could see into the antrum, as all the bone was destroyed, and I think he has had grand results."

DR. HANCOCK: "I have seen one other case of this kind. It was operated upon by Dr. Wyeth, of New York. It was not so bad as this, but the entire surface had dropped in. He used a metal support and inserted it into the nose as a kind of brace. This remained two or three months, but had sloughing, and it was removed.

DR. DIVINE: The most interesting thing to me about this case is the fact that he had union to take place after having the patient under treatment only a few months. Affected as she was, I think the results are very fine indeed. Although the nose is not very good looking, yet the results are good

DR. NOBLE: I would say that I operate at the hospital on syphilitic cases after short treatment and I do not have any trouble in getting union.

DR. MCRAE: I have a case here in my pocket which to me is a remarkable one. An old gentlemen sixty-six years of age with a typical history of enlarged prostate. He has been under my care for about four weeks. I have been washing out his bladder and he has the largest amount of residual urine of any case I have seen. He had from a pint and a half to two pints. He did notimprove under treatment at all. I did not examine him for stone in the bladder, as he had enlarged prostate, which seemed to be the entire cause of his trouble. I operated upon him and found the most remarkable specimen I have found anywhere. It looks like a lot of jack-stones glued together. I have submitted a piece of the specimen to Dr. Bourns for examination to see what he can make out of it. There is no pocket, and why it assumed that shape I can not say; it looks more like a coral growth or a sweet-gum burr. It was perfectly loose in the bladder, had no coating around it, and was perfectly black when it came out. The median lobe of the prostrate was enlarged and simply filled up the space. I operated to-day and the patient seems to be doing well.

DR. STIRLING: About two weeks ago I had a somewhat peculiar case. A man from the country had a tooth pulled by a traveling dentist and a few days later complained of symptoms of antrum disease which I treated and decided to operate on, but just before the time for so doing he came to me with the root of a tooth in his hand and in high glee. The root had escaped from the antrum into his nose, from which he expelled it by sneezing, and the antrum trouble then promptly disappeared.

DR. MURPHY: I had a wisdom tooth which gave me a great deal of trouble last spring, and I went to a dentist to have it extracted. He used a hypodermic injection and extracted the tooth. I went home and two hours afterward had a chill and the next day had a fever and the next day another chill. I was sick from the effects of it for three or four months. In about eight weeks I had an eruption come out all over my body; it was very similar to that of measles and went all over the body. After this eruption I felt some better, but it affected

3

me more or less all the summer. I suppose it was due to septic poisoning.

DR. NOBLE: I think it would be a good idea for some smart member, who can write well, to get up the facts in regard to neglect of aseptic precautions by the dentists and write them out.

DR. MCRAE: In this case Dr. Murphy came very near losing his life. I saw him, and his pharynx was œdematous. It was a case of typical septic poisoning. I think the dentist not only used a dirty syringe, but that he used a solution into which he had evidently before been dipping the dirty syringe. We have the grossest neglect of asepsis and I have had three cases of chancre as the result of the use of dirty instruments by dentists.

DR. BENSON: I had an experience along the same line in a dentist's office the other day. I was called in to give ether to a lady to have her teeth extracted. The operation was quite extensive, but the dentist made no preparation whatever, but pulled the teeth, opened up abscesses, etc., and laid the instruments down and used them again without any care of preparation. I think this should be taken in hand; I believe people lose their lives by such carelessness. I know a lady who was sick over two months all on account of being treated in a way similar to Dr. Murphy.

DR. LOVE: Like Dr. McRae, I have my case with me, but is of a different character and is not in very good condition for inspection at present. I would like some suggestions from the Society in regard to it. It is the second time that such a thing has occurred to me. I had occasion the other day to use some bromine and in endeavoring to get it out of the bottle it exploded and went all over my hand and burnt it very much. I have treated this as I did before, simply as a burn, but it does not seem to yield to the treatment.

DR. MCRAE: I do not know any reason why it should be treated different from an ordinary burn, as you have destruction of the tissue there. I would treat it on general principles of asepsis as applied to burnt or lacerated surfaces. If the surface is large I would have skin-grafting, as the hand is exposed, and this would prevent contraction and cracking.

DR. DUNCAN: If Dr. Love will take one part of gum camphor to four parts of vaseline and keep it applied to the surface, it will prevent scarring and cracking to a great extent.

DR. VISANSKA: In regard to burns by bromine, I would mention first the prophylaxis, and that is to place the bottle in ice water before attempting to open it, and this will prevent any explosion. After a person is burned with the bromine I would immediately use some soda solution so as to get the formation of some bromide of soda, and after this would use antiseptic dressing.

DR. LOVE: As soon as it happened I washed it in a strong soda solution and then applied a prescription which I have used in treating burns very successfully, but I do not seem to have perfect results in this instance.

DR. LONGINO: In regard to burns, the worst burn I ever saw was when I was a student. A little girl was burned to death and her two aunts had their hands terribly burned in attempting to smother the flames. I remember an old physician treated these two cases and he simply put on some flour and wrapped them up and kept the dressings saturated with sweet oil. The results were as good as I have ever seen. They were the worst of burns, and one of the ladies lives in Atlanta and her hands have no scars on them at all.

DR. MCRAE: As there seem to be no other reports, I would state in this connection that there was a very interesting paper read at the surgical section of the American Medical Association on the subject of skin-grafting. The gentleman had been making experiments along this line for a number of months and perhaps years, and instead of using the fresh skin he would use simply the epidermis which had been obtained from the skin previously by vesication. The specimens of epidermis which he presented were taken from a man who fell into a vat of hot brine. He said that where they are kept perfectly dry they can be used seven or eight months after they have been removed. His results seemed to be most excellent. It is a very simple and effectual method of skin-grafting, and by it the large scars and ulcers can be removed. You can easily tell the outer from the inner surface of the epidermis, for it always curls inward.

DR. HANCOCK: Dr. Jarnagan related a case to me last year of a man who was injured at the *Journal* office and upon whom the shoulder amputation was made, and in grafting he used scrapings of the epidermis from the heel and had most excellent results.

DR. NOBLE: I will mention a case which had been operated upon a week before I saw it. It was an abdominal operation. The stitches had been removed, and in coughing the abdomen came open. The operation had been performed perfectly, but it seems that in some way a piece of the omentum had gotten in the wound and thus prevented perfect adhesion, and so when the stitches were removed and the patient coughed, the wound opened. The skin and everything gave way and the bowels came out under the sterile dressing. I had the patient on the table at the time, and I simply closed the wound and the patient did not have any rise of temperature, but got along all right. I had never seen one before which came open all the way through; I had seen them come loose between the skin, but not through all the tissues.

MISCELLANEOUS BUSINESS.

Dr. Hancock stated that his committee had expended twenty dollars, ten dollars for witnesses and ten dollars additional to prescriptions gotten by this witness toward the evidence in pay for prosecuting the unregistered doctors.

Dr. McRae moved that this amount be appropriated for the use of the committee, and that the Society indorse the action of the committee. Motion carried.

Dr. Hancock called attention to the fact that the attorneys had secured evidence against the assistants of some of the regular physicians, and that the attorneys would prosecute them unless they were otherwise instructed by the society; that the committee had not been empowered to use its discretion in such cases.

After a discussion of this matter Dr. McRae moved that the committee be empowered to investigate all such cases and to use their discretion in prosecuting them. Motion carried.

The question of suspending the meetings of the society was brought up, and upon vote it was decided to continue them through the summer months.

Drs. J. C. Olmstead, F. W. McRae and L. H. Jones were appointed as committee to look into the matter of changing the Constitution.

In regard to printing the minutes of the society, it was moved and carried that they be offered to our local journals, but if the society deemed fit at any time they could send them to other journals.

